# Heat Shock Proteins Regulatory Role in Neurodevelopment

**DOI:** 10.3389/fnins.2018.00821

**Published:** 2018-11-12

**Authors:** David J. Miller, Patrice E. Fort

**Affiliations:** ^1^Department of Ophthalmology and Visual Sciences, University of Michigan, Ann Arbor, MI, United States; ^2^Department of Molecular and Integrative Physiology, University of Michigan, Ann Arbor, MI, United States

**Keywords:** heat shock proteins, neurodevelopment, neurite extension, cell migration, axon guidance, neurovascular unit

## Abstract

Heat shock proteins (Hsps) are a large family of molecular chaperones that are well-known for their roles in protein maturation, re-folding and degradation. While some Hsps are constitutively expressed in certain regions, others are rapidly upregulated in the presence of stressful stimuli. Numerous stressors, including hyperthermia and hypoxia, can induce the expression of Hsps, which, in turn, interact with client proteins and co-chaperones to regulate cell growth and survival. Such interactions must be tightly regulated, especially at critical points during embryonic and postnatal development. Hsps exhibit specific patterns of expression consistent with a spatio-temporally regulated role in neurodevelopment. There is also growing evidence that Hsps may promote or inhibit neurodevelopment through specific pathways regulating cell differentiation, neurite outgrowth, cell migration, or angiogenesis. This review will examine the regulatory role that these individual chaperones may play in neurodevelopment, and will focus specifically on the signaling pathways involved in the maturation of neuronal and glial cells as well as the underlying vascular network.

## Introduction

Heat shock proteins (Hsps) are a large family of evolutionarily conserved molecular chaperones with pivotal roles in cell survival and development. Hsps can be broadly classified into two families based on comparable molecular mass. First, the small, ATP-independent Hsps are chaperones of a molecular mass between 8 and 28 kDa. These chaperones include ubiquitin, the α-crystallins, HspB1 (also known as Hsp25 in mice or Hsp27 in rats and humans), and many others (for review, see Bakthisaran et al., 2015). These small Hsps have received increasing attention in recent years, mostly due to their potential in protective approaches. Second, the large, ATP-dependent Hsps are chaperones of a molecular mass between 40 and 105 kDA. These include the well-known chaperones of the 70 and 90 kDa families. The 70 kDa group consists of the stress-inducible Hsp70 and the constitutively expressed heat shock cognate 70 (Hsc70). Similarly, the 90-kDa group consists of two major isoforms, namely the inducible Hsp90α and the constitutively expressed Hsp90β. However, because these isoforms are often difficult to isolate, many studies have resorted to studying co-purified aggregates containing both Hsp90α and Hsp90β, simply referring to the whole as Hsp90 (Sreedhar et al., 2004). Other large Hsps include the Hsp40s or J-proteins, which interact with Hsp70 through their J domain and serve as regulatory co-chaperones (Walsh et al., 2004).

Hsps confer thermotolerance in all organisms in which they have been studied (Lindquist and Craig, 1988), but also provide protection from insults such as hypoxia and cytotoxic exposure. Initially discovered as a group of proteins upregulated in heat-stressed *Drosophila melanogaster* (Ritossa, 1962; Tissières et al., 1974), Hsps are now understood to perform critical functions both in stressed and “unstressed” conditions (i.e., in the absence of supraphysiologic stress). The protective effects of Hsps are mediated at least in part through their chaperone functions. Molecular chaperones are proteins that facilitate native protein stabilization, translocation, re-folding, and degradation. These functions are often performed with the assistance of co-chaperones, which regulate chaperone affinity for a given substrate. Together, Hsps and their respective co-chaperones not only ensure protein quality control, but also prevent protein aggregation that would otherwise overwhelm the cell and lead to programmed cell death or necrosis. Despite these functional similarities, individual Hsps vary considerably in their expression, protein structure, localization, and ability to be induced (Table 1).

**Table 1:** Nomenclature, function, and distribution of heat shock proteins.

Gene symbol	Description	Stress-inducible	Localization^∗^	Reference
*HSPB1*	HspB1; Hsp25 (mice); Hsp27 (rats, humans)	+	Brain, retina, spinal cord, sciatic nerve	Dean and Tytell, 2001; Kirbach and Golenhofen, 2011
*HSPB2*	HspB2; MKBP	-	Brain, sciatic nerve	Kirbach and Golenhofen, 2011
*HSPB3*	HspB3	-	Brain, spinal cord, sciatic nerve	Kirbach and Golenhofen, 2011
*HSPB4*	HspB4; αA-crystallin	+	Retina, sciatic nerve	Xi et al., 2003; Kirbach and Golenhofen, 2011; Ruebsam et al., 2018
*HSPB5*	HspB5; αB-crystallin	+	Brain, retina, spinal cord, sciatic nerve	Bhat and Nagineni, 1989; Xi et al., 2003; Kida et al., 2010; Kirbach and Golenhofen, 2011
*HSPB6*	HspB6; αC-crystallin	-	Brain, spinal cord, sciatic nerve	Kirbach and Golenhofen, 2011
*HSPB7*	HspB7	-	Sciatic nerve	Kirbach and Golenhofen, 2011
*HSPB8*	HspB8; Hsp22; H11 kinase	+	Brain, spinal cord, sciatic nerve	Kirbach and Golenhofen, 2011; Ramirez-Rodriguez et al., 2013
*HSPB9*	HspB9	Unknown	Testis	Kappé et al., 2001
*HSPB10*	HspB10	Unknown	Testis	Fontaine et al., 2003
*HSPB11*	HspB11	-	Brain, spinal cord, sciatic nerve	Kirbach and Golenhofen, 2011
*HSPA1A, B*	Hsp70 family A, members 1A and 1B	+	Brain, retina, spinal cord	Manzerra and Brown, 1992; Tytell et al., 1994; D’Souza and Brown, 1998; Dean et al., 1999; Loones et al., 2000
*HSPA8*	Hsc70	-	Brain, retina, spinal cord	D’Souza and Brown, 1998; Dean et al., 1999; Loones et al., 2000; Chen and Brown, 2007
*HSP90AA1*	Hsp90α family class A, member 1	+	Brain, retina, spinal cord	Ishimoto et al., 1998; Loones et al., 2000; Bernstein et al., 2001; Zuehlke et al., 2015
*HSP90B1*	Hsp90β family member 1; Gp96; Grp94	-	Brain, retina, spinal cord	Ishimoto et al., 1998; Loones et al., 2000; Bernstein et al., 2001
*HSPH1*	Hsp105	+	Brain, dorsal root ganglion	Hatayama et al., 1997; Saito et al., 2007

*^∗^Not an exhaustive list. Special attention was given to the distribution of heat shock proteins in the nervous system.*

One of the essential roles of Hsps under “normal” conditions is to promote proper embryonic and postnatal development of multiple organ systems, particularly the nervous system (Gershon et al., 1990; Walsh et al., 1997; Luo et al., 2006; Mimura et al., 2007; Patterson and Zhang, 2010). During embryonic development, neuronal and glial progenitors must survive a relatively hypoxic microenvironment, and simultaneously take on energetically expensive endeavors such as neurite outgrowth and cell migration. These events must occur in concert so that neurons can form the appropriate connections and receive support from the nearby glia and microvasculature. Independent of development, such challenging environments are strongly associated with the stress-inducible Hsps, suggestive of a potential role for these proteins also during neurodevelopment. Indeed, recent studies have uncovered that individual Hsps directly regulate neurodevelopment itself through modulation of pathways involved in cell growth and migration, such as the PI3K/Akt and RhoA signaling cascades (Konishi et al., 1997; Sato et al., 2000; Hennessy et al., 2005; Dou et al., 2007; Banz et al., 2009; Wang et al., 2012; Sun et al., 2013; Benitez et al., 2014).

Here, we review the regulatory role of Hsps in embryonic and postnatal neurodevelopment. After an overview of the spatio-temporal expression of Hsps during embryogenesis, the specific signaling pathways by which these chaperones regulate neuronal and glial differentiation and migration are examined. Finally, this review concludes with the contribution of these chaperones to the development of the neurovascular unit. The studies presented herein demonstrate that Hsps are not simply pro-survival factors expressed during cellular stress. Rather, Hsps are also critical mediators of cell growth and migration, axon guidance, and angiogenesis (Frebel et al., 2007; Kase et al., 2010; Colvin et al., 2014; DeGeer et al., 2015; Li et al., 2015; Shimizu et al., 2016).

## Heat Shock Protein Expression and Regulation During Neurodevelopment

### Heat Shock Protein Expression During Embryonic Neurodevelopment

Numerous studies have shown that individual Hsps have specific patterns of expression during embryonic neurodevelopment. Walsh et al. (1997) performed extensive work on cultured rat embryos and showed that the expression of inducible and constitutive Hsps is tightly regulated at critical steps in early embryogenesis, particularly during neural plate induction at E9.5. Specifically, they showed that expression of Hsp90 and Hsp70 is correlated with the cell cycle, with Hsp90 being highly expressed between G_0_-G_1_, whereas Hsp70 is most highly expressed between G_2_-M. More expectedly, they showed that these patterns are altered with heat shock treatment. Differential expression of these proteins in untreated and heat-stressed neuroectodermal cells suggests distinct roles of these proteins in neurodevelopment. Subsequent research has extended this work by examining regulation of Hsps in later stages of embryogenesis. In the mouse brain, the small Hsp, HspB1, is closely associated with cortical neurons and radial glia undergoing differentiation at E12.5 (Loones et al., 2000). In addition, HspB1 is strongly associated with the first endothelial cells originating from the neural crest cells. Constitutively expressed Hsps, namely Hsc70 and Hsp90β are present at high levels during this developmental stage, whereas their inducible counterparts, Hsp70 and Hsp90α, are not seen until E15.5. These findings are consistent with a previous study conducted by Tanaka et al. (1995), and suggest that the ATP-dependent inducible Hsps may have greater importance in the later stages of embryonic development. Late embryonic development is also characterized by a decline in constitutive expression of Hsc70 in the hippocampus (Hatayama et al., 1997), further supporting the hypothesis that inducible Hsps may have a relatively greater contribution during the final stages of embryonic neurodevelopment, when oxidative stress is also increased.

The retina, an extension of the central nervous system (CNS), is an excellent tissue to study neurodevelopment. Using *in situ* hybridization, Tanaka et al. (1995) characterized the mRNA expression of large Hsps during embryonic and postnatal development of the retina and other ocular tissues. While certain Hsps, such as Hsp70, were not detectable, others exhibited a specific pattern of expression. The authors showed that Hsc70 is expressed in the cornea, lens, choroid, sclera, and neuroectoderm during early embryogenesis. Toward mid to late gestation (i.e., E15.5-E16.5), Hsc70 expression is downregulated in most ocular tissues, but not the retina. In the retina, Hsc70 expression persists into adulthood. Similarly, Hsp86 (murine homolog of Hsp90α) expression persists exclusively in the retina. Subsequent studies showed that Hsc70 has a specific pattern of expression in chick retinal neurogenesis (Morales et al., 1998). While Hsc70 is initially expressed in most neuroepithelial cells, this expression becomes restricted to a subset of these cells in the peripheral retina as development proceeds. Those cells identified as retinal ganglion cells (RGCs), the first neurons to differentiate in the retina, continue to exhibit Hsc70-positivity throughout development. In these cases, continued Hsc70 expression may be necessary due to the absence of Hsp70.

### Heat Shock Proteins Continued Role in Postnatal Neurodevelopment

Differential expression of Hsps continues into the postnatal period, during which neurons and glia continue to differentiate and migrate to their final destinations. As mentioned previously, Hsp70 and Hsp90α are first detectable in the mouse brain toward mid to late gestation (i.e., E15.5) (Tanaka et al., 1995; Loones et al., 2000). In contrast, Hsc70 expression begins to decline in the chick retina and mouse hippocampus at this time (Hatayama et al., 1997; Morales et al., 1998). In the postnatal period, however, Hsc70 expression does not simply continue to decline but remains relatively constant throughout all regions of the rat brain, from the cerebrum to the brainstem and cerebellum (D’Souza and Brown, 1998; Table 1). On a subcellular level, Hsc70 is specifically localized in the perikarya and apical dendrites of Purkinje cells and deep cerebellar neurons from P1 into adulthood. Similarly, Hsp90 expression remains relatively constant, and shares a common subcellular localization in Purkinje cells throughout the postnatal period and into adulthood. The continued spatio-temporal regulation of Hsps suggests that these chaperones remain important in the later stages of CNS development. Given their subcellular localization, Hsc70 and Hsp90 may also contribute to processes such as neurite outgrowth, which will be discussed in a subsequent section of this review.

### Small Heat Shock Proteins in Embryonic and Postnatal Neurodevelopment

By mid- to late-stage gestation, many of the small Hsps are detectable in one or more structures of the mammalian brain, each with their own individual pattern of expression (Table 1). In the rat hippocampus, several small Hsps, including HspB1 and HspB8, are expressed at low basal levels at E17-E19 (Kirbach and Golenhofen, 2011). Expression of these chaperones remains low in the early postnatal period, but is significantly increased by 9–10 weeks, suggesting a temporally regulated role, at least in the hippocampus. While HspB1 has been implicated in processes such as neuronal differentiation and neurite extension, less is known about the specific function of HspB8. Recent studies have indicated that this chaperone may play a key role both in neurogenesis and neurodegenerative diseases. Ramirez-Rodriguez et al. (2013) showed that overexpression of HspB8 promotes differentiation and survival of dentate gyrus precursor cells *in vitro*. The same authors also showed that removal of the highly conserved α-crystallin domain abrogates HspB8-mediated differentiation and survival. In addition, HspB8 mutations are associated with Charcot-Marie-Tooth disease, a neurodegenerative disease characterized by demyelination or impaired axon transport (Gentil and Cooper, 2012). Similar to HspB8, relatively little is known about the role of other small Hsps such as the α-crystallins. However, it has been postulated that the α-crystallins could play an important role in neurodevelopment since their expression is regulated by the transcription factor Pax6, the “master regulator” of brain and eye development (Piri et al., 2016). A role of α-crystallins in neurodevelopment is supported by the finding that Pax6 regulates the survival of adult dopaminergic olfactory bulb neurons via αA-crystallin (Ninkovic et al., 2010). Given that Pax6 is even more abundantly expressed during embryonic CNS development (Duan et al., 2013), this finding strongly suggests that Pax6 and αA-crystallin may also regulate the survival of neuronal progenitors. Further evidence for a role of α-crystallins in neurodevelopment can be seen in the work of Shao et al. (2013) who showed that the anti-inflammatory properties of αB-crystallin are regulated at least in part by the astrocytic dopamine D2 receptor. Importantly, the astrocytic D2 receptor is expressed in the corpus striatum during embryonic development (Lin et al., 2001), raising the possibility that this receptor and its downstream target, αB-crystallin, could suppress neuroinflammation and promote cell survival in the early stages of neurodevelopment. However, the astrocytic dopamine D2 receptor also regulates GSK3β, which is known to regulate neuronal cell growth, polarity, proliferation, and survival (Cui et al., 1998; Gärtner et al., 2006). Because of these overlapping functions, the exact role of astrocytic dopamine D2 receptor remains unclear; however, it is likely to involve αB-crystallin. In addition to regulation by Pax6 and membrane-bound receptors, embryonic and postnatal expression of the α-crystallins may also be regulated by one or more heat shock factors (HSFs), which will be discussed next.

### Essential Regulatory Role of Heat Shock Factors During Neurodevelopment

Gene knockout studies have highlighted a requirement of certain Hsps during neurodevelopment. During early embryogenesis, the developing CNS is particularly sensitive to heat shock, and prolonged heat exposure is associated with neural tube defects as well as microphthalmia (Walsh et al., 1997). In the postnatal period, Hsps continue to protect the developing CNS from unfavorable conditions. Hsps are also important in unstressed conditions, during which the stress-inducible Hsps are transcribed at low basal levels. Expression of Hsps is regulated at the transcriptional level by factors known as HSFs. These factors promote transcription by interacting with adjacent or intronic heat shock elements (HSEs). HSF1 and HSF2 promote transcription of numerous genes, including HspB1, Hsp40, Hsp70, Hsp90α, Hsp90β, and other non-Hsp genes such as p35 (Trinklein et al., 2004; Åkerfelt et al., 2007; Östling et al., 2007; Metchat et al., 2009). HSF1, in particular, is critical to proper neurodevelopment, which is demonstrated in HSF1^−/−^ mice that have impaired olfactory neurogenesis (Takaki et al., 2006), as well as impaired hippocampal spinogenesis and neurogenesis (Uchida et al., 2011). Consistent with the spatio-temporal expression of Hsps described above, there is a narrow time-window in the neonatal period during which restoration of HSF1 can rescue hippocampal development. Restoration of HSF1^−/−^ in neonates with a vector overexpressing constitutively active HSF1 Rephrased for clarity; see previous comment rescues the phenotype, whereas the same treatment in adults confers no such benefit. These findings strengthen the argument that Hsps have a critical, time-dependent role during normal neurodevelopment. However, because HSF1 regulates transcription of many proteins, it has been difficult so far to narrow down which of its targets are most essential.

HSF2 is highly homologous to HSF1 and shares overlapping DNA-binding activity at certain HSEs (Trinklein et al., 2004; Åkerfelt et al., 2007; Östling et al., 2007); however, HSF2 is not clearly involved in the temporally-regulated expression of Hsps (Loones et al., 1997). This is supported by the fact that HSF2 cannot compensate for the loss of HSF1 function in heat-stressed HSF1^−/−^ mouse embryonic fibroblasts *in vitro* (McMillan et al., 1998). Nevertheless, HSF2 appears to play complex regulatory roles in neurodevelopment. Although one study reported that HSF2^−/−^ mice have normal brain architecture and cognitive development (Mcmillan et al., 2002), several others have since made opposite observations. Those subsequent studies indeed reported that HSF2^−/−^ mice have neurodevelopmental or reproductive abnormalities, namely ventricle enlargement and reduced viability of pachytene spermatocytes (Kallio et al., 2002; Wang et al., 2003, 2004; Chang et al., 2006). Such opposing results were suggested to be due to differences in the knockout model or genetic background, the latter of which could alter penetrance of the phenotype (Mcmillan et al., 2002). Subsequently, Chang et al. (2006) showed that neither the reduced number of radial glia and radial glia-derived astrocytes nor the impaired neuronal migration in HSF2^−/−^ mice were directly associated with Hsps. Rather, these changes were associated with reduced p35-Cyclin-dependent kinase 5 signal transduction. While a role of HSF2 cannot be totally ruled out, these data suggest that Hsps rely primarily on HSF1 during embryogenesis.

## Heat Shock Proteins Potential Role in Neuronal and Glial Differentiation

### HspB1 Expression Is Correlated With Neuronal Differentiation

During neurodevelopment, HspB1 exhibits a specific spatio-temporal pattern of expression that coincides with neuronal differentiation. In the CNS, neural tube closure and the appearance of the first neurons occurs at E8.5, whereas HspB1 expression is first detectable shortly thereafter (Easter et al., 1993; Walsh et al., 1997; Loones et al., 2000). In mice, HspB1 is relatively abundant in specific brain regions, including the zona limitans interthalamica as well as the axons that comprise the peripheral and longitudinal tracts. Between E12.5 and E15.5, HspB1 synthesis is further enriched in regions such as the olfactory bulbs and corpus striatum. This specific pattern of expression suggests that HspB1 is associated with neuronal differentiation. A key role of HspB1 in neuronal differentiation is further supported by the finding that this chaperone is upregulated in human embryonic stem cell-derived differentiating motor neurons, but subsequently downregulated following maturation, all of which in the absence of additional stressful stimuli (Chaerkady et al., 2009). Similarly, in the developing mouse brain, HspB1 is globally downregulated after the first round of neuronal differentiation (i.e., E12-E16) (Cheng et al., 2016). This reduced expression of Hsps during maturation is consistent with *in vitro* studies suggesting that differentiation reduces neuronal cells’ resistance to stress. Indeed, Hsp60 and Hsp70 are not as readily induced in differentiated neuronal phaeochromocytoma (PC12) cells in the setting of hyperthermia or cytotoxic exposure (Dwyer et al., 1996). Similarly, several Hsps, including HspB1, are less readily induced in differentiated neuroblastoma-glioma hybrid cells (Oza et al., 2008). Together, these studies are consistent with the notion that that neuronal differentiation regulates basal and inducible Hsp expression. Interestingly, there is growing evidence that the reverse is also true; Hsps influence neuronal differentiation.

### Heat Shock Proteins Complex Regulatory Role in Neuronal Differentiation

**FIGURE 1:**
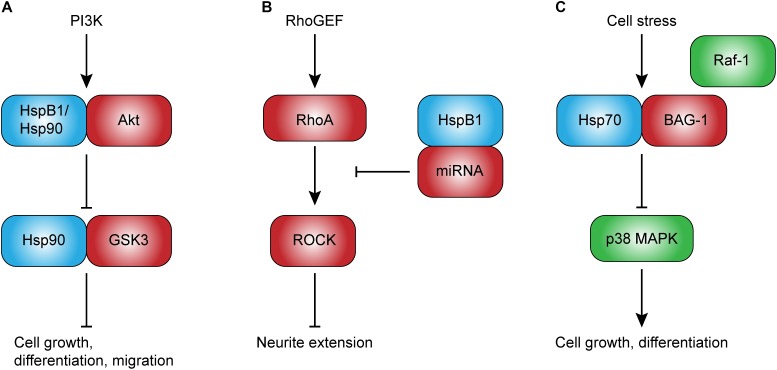
Potential intracellular roles of heat shock proteins in development. Intracellular Hsps, such as HspB1 and Hsp90, directly regulate client proteins involved in cell growth and migration **(A)**, and may play an important role in neurodevelopment. HspB1, in particular, may also regulate neurite extension through the upregulation of non-coding RNAs that inhibit RhoA-ROCK signaling **(B)**. Other Hsps, such as Hsp70, have been shown to competitively inhibit Raf-1-BAG-1-mediated cell growth and differentiation **(C).** This novel role of Hsp70 may be especially important given its ability to be rapidly induced. Abbreviations: PI3K, phosphoinositide 3-kinase; GSK3, glycogen synthase kinase 3; BAG-1, Bcl2-associated anthogene-1; RhoGEF, Ras homolog guanine exchange factor; RhoA, Ras homolog gene family, member A; ROCK, Rho-associated protein kinase.

The requirement of HSF1 for embryogenesis, coupled with the diverse chaperone functions of Hsps, suggests that Hsps could mediate cell differentiation. Recent research has shown that prolonged heat exposure promotes differentiation and proliferation of hippocampus-derived neuronal, and, to some extent, glial progenitor cells (Matsuzaki et al., 2009). The same group also showed that even mild heat exposure promotes differentiation of hippocampal neuronal progenitor cells, and that this increase in differentiation is accompanied by changes in Hsp mRNA expression (Hossain et al., 2017). In particular, HspB1 expression increases in neuronal progenitors undergoing differentiation, whereas Hsp70 and Hsp90 expression declines. As the authors noted, HspB1 may promote differentiation through its chaperone interactions with the client protein Akt (protein kinase B; Figure 1A), which is central to cell growth and survival (Konishi et al., 1997; Hennessy et al., 2005). However, Akt also interacts with Hsp90, suggesting a complex regulation of its function through dynamic interaction with Hsps during differentiation.

Consistent with this complex dynamic of protein–protein interaction of Hsps with specific client proteins, recent studies have also suggested that a non-specific reduction in Hsp expression may promote differentiation. Hsps are regulated at the transcriptional level by the transcription factor HSF1, which, in turn, is regulated at the protein level by various stressors and negative feedback (for review, see Anckar and Sistonen, 2011). HSF1 is also regulated by nicotinamide adenine dinucleotide (NAD)-dependent deacetylase sirtuin-1 (SIRT-1) (Westerheide et al., 2009). Knockdown of SIRT-1 was recently reported to promote neuronal differentiation in rat embryonic and N2a progenitor cells *in vitro* (Liu et al., 2014). Of note, the authors reported that increased neuronal differentiation is associated with a reduction in Hsp70. While this finding is consistent with a role of Hsps in neuronal differentiation, it is important to note that HSF1 regulates transcription of numerous Hsp and non-Hsp genes (Trinklein et al., 2004; Metchat et al., 2009). Knockdown of SIRT-1, therefore, likely results in a non-specific, widespread reduction of chaperone activity and an accumulation of denatured proteins. Hsps not only manage compromised proteins, but also regulate protein maturation and stability. Thus, reduced Hsp expression associated with SIRT-1 knockdown could affect the stability of specific targets critical for neuronal differentiation. Because this study focused exclusively on Hsp70, subsequent studies are necessary to examine other Hsps, including HspB1, to help elucidate the exact role of stress-inducible Hsps in neuronal differentiation.

### Hsp90 Is Necessary for Neuronal Polarization and Axon Specification

Hsp90 is known to be critical for spermatogenesis and embryonic development (Voss et al., 2000; Grad et al., 2010), and due to its ubiquitous expression in the CNS, is likely to play an important role in neurodevelopment as well. Over the past two decades, several studies have shown that Hsp90 is correlated with neuronal differentiation. Loones et al. (2000) reported that the inducible isoform of Hsp90, Hsp90α, is first detectable in the developing mouse brain at E15.5, several days after the first neuronal and glial progenitors begin to differentiate. Several others have confirmed that Hsp90 expression is correlated with neuronal differentiation *in vitro*. Specifically, Hsp90 is markedly increased in neuronal P19 cells and embryonic hippocampal neurons undergoing differentiation (Quintá et al., 2010; Afzal et al., 2011). Subsequent experiments showed that Hsp90 becomes associated with the cytoskeleton of differentiating hippocampal neurons, particularly in branch points and terminal ends (Quintá and Galigniana, 2012). These findings suggest that intracellular Hsp90 could modulate cytoskeleton dynamics and key developmental events such as neuronal polarization.

Neuronal polarization is the process by which a neuron establishes an axon and dendrites, and is a critical step in neuronal differentiation. Interestingly, one recent study has shown that Hsp90 is required for key events in neuronal polarization. Using neurons isolated from moues embryos at E17, Benitez et al. (2014) showed that pharmacologic inhibition of Hsp90 with the Hsp90 inhibitor, 17-demethoxygeldanamycin (17-AAG), both disturbs neuronal polarization and slows axon elongation. In addition, these perturbations were accompanied with a reduction in PI3K/Atk/GSK3 signal transduction. The PI3K/Akt/GSK3 signaling cascade is well-known to mediate cell growth, differentiation, proliferation, migration, and even apoptosis (Gregory et al., 2003; Hennessy et al., 2005). Of note, both Akt and GSK3 are client proteins for Hsp90 (Sato et al., 2000; Dou et al., 2007; Banz et al., 2009), immediately suggesting a possible mechanism by which Hsp90 could regulate functions such as cell differentiation in the context of normal neurodevelopment (Figure 1A). An interesting finding in aforementioned work is that inhibition of Hsp90 was associated with an increase in Hsp70 and Hsc70, which the authors noted may reflect a compensatory response in an attempt to maintain signal transduction. Besides the regulation of signal transduction cascades, Hsp90 has been implicated as a paracrine factor promoting dendrite-like outgrowth and differentiation *in vitro* (Park et al., 2015). A role for extracellular Hsps, including Hsp90, has also been suggested, and will be discussed in a subsequent section of this review.

### Heat Shock Proteins in Differentiating Glia

Although there is limited research on the role of Hsps in differentiating glia, Hsps are expressed and induced in mature glia (Krueger et al., 1999; Dean and Tytell, 2001; Kalesnykas et al., 2008), and our group and others have recently suggested that Hsps play an important role in these cells (Ruebsam et al., 2018). In addition to their critical house-keeping functions and pro-survival role in mature cells under stress, Hsps also exhibit specific patterns of expression during glial development. For instance, in human fetal telencephalon, αB-crystallin is expressed in numerous radial glia, a subset of oligodendrocyte progenitors, and a small number of astrocytes (Kida et al., 2010). Similar to what has been reported in neurons, the ability to induce Hsps synthesis in mature glia is reduced compared to progenitor cells (Zhang et al., 2001). These data strongly suggest tightly regulated roles for Hsps in glial development. Because neurons and glia are intimately connected, it is possible that synthesis of Hsps in glia may regulate adjacent neuronal cells’ growth. Some researchers have already demonstrated this phenomenon *in vitro*, and their findings will be reviewed here in subsequent sections.

## Heat Shock Proteins Regulate Neuronal and Glial Cell Growth

### Small Heat Shock Proteins Promote Neurite Outgrowth and Extension

HspB1 is primarily known for its role in thermotolerance and cytoprotection (Arrigo and Welch, 1987; Lavoie et al., 1993a; Bartelt-Kirbach et al., 2017), but recently it has been implicated as a potential contributor to neurodevelopment. The first evidence that this chaperone not only promotes cell survival, but also axonal growth, came from a study showing that HspB1 is rapidly upregulated in response to peripheral nerve injury and promotes its regeneration (Costigan et al., 1998). The role of HspB1 in the regulation of neurite outgrowth has since been further characterized. In the early stages of neurite growth initiation, HspB1 is mainly localized in lamellipodia and focal adhesions, whereas in the later stages of neurite extension, HspB1 is found in branch points, processes, and growth cones (Williams et al., 2005), suggesting a role in the promotion of growth by modulation of cytoskeletal dynamics. This role in neurite outgrowth has been subsequently confirmed by studies showing that overexpression of HspB1 enhances neurite extension and branching, whereas inhibition with short interfering ribonucleic acid (siRNA) reduces neurite length and complexity (Williams et al., 2006; Ma et al., 2011).

Despite strong evidence for a role of HspB1 in neurite outgrowth and extension, the exact mechanisms remain unclear. HspB1 is well-known to interact with the cytoskeleton in a phosphorylation state-dependent manner (Lavoie et al., 1993b; Guay et al., 1997; Pichon, 2004), leading some to hypothesize that this chaperone could mediate cell growth through direct modulation of cytoskeleton dynamics (Lavoie et al., 1993a; Costigan et al., 1998). This hypothesis is supported by the observation that HspB1 co-localizes with actin in the early stages of neurite initiation (Williams et al., 2005). However, the significance of this interaction in terms of neuronal cell growth, has not been demonstrated. Another possible mechanism is that HspB1 regulates cytoskeletal dynamics through signal transduction cascades. One study supporting this hypothesis showed that HspB1 indirectly inhibits RhoA signal transduction by upregulating non-coding RNAs that inhibit Rho guanine exchange factor 11 (Figure 1B; Sun et al., 2013). RhoA is a small GTPase known to inhibit neurite extension through Rho-associated protein kinase (ROCK)-mediated inhibition of the actin cytoskeleton (Bito et al., 2000). Thus, HspB1 may promote neurite extension through inhibition of this negative feedback loop.

αA- and αB-crystallin are two other members of the small Hsps initially thought to be present exclusively in the crystalline lens, but now understood to be expressed in numerous tissue types, including the brain, retina, heart, and skeletal muscle (Taylor and Benjamin, 2005; Bakthisaran et al., 2015). While their roles in retinal neuroinflammation and neuroprotection have been clearly demonstrated (for review, see Dulle and Fort, 2016; Rübsam et al., 2018), their contribution to neuronal cell growth is still under investigation. Wang et al. (2011) showed that α-crystallins can promote neurite initiation and extension of retinal ganglion cells (RGCs) grown on inhibitory myelin substrate in a dose-dependent manner. The same group subsequently demonstrated that intravitreal injection of α-crystallins significantly enhances axonal regeneration following optic nerve crush in newborn rats (P0-P2) (Wang et al., 2012). Furthermore, this enhanced axonal regeneration was associated with a reduction in the protein activity of RhoA/ROCK, consistent with the above suggested mechanism of increased neurite extension. An important limitation of these studies is that the researchers studied co-purified aggregates containing αA- and αB-crystallins, precluding at this point a clear understanding of the relative contribution of each individual chaperone. Starting to clarify the individual roles of αA- and αB-crystallin, a recent study reported an association between αA-crystallin, inhibition of astrocyte activation, and axon elongation following optic nerve crush (Shao et al., 2016). On the other hand, cell culture experiments using hippocampal neurons have shown that αB-crystallin regulates dendritic complexity, and does so in a phosphorylation-dependent manner (Bartelt-Kirbach et al., 2016). In addition, recent reports suggest that the small Hsps could mediate neurite extension not only through modulation of microtubule assembly (Ghosh et al., 2007), but also as a guidance cue. Before further examining these mechanisms and a possible extracellular role of Hsps, this review will focus on the potential role of other Hsps in cell growth.

### Hsp70 and Hsp90α Regulate Cell Growth Through Their Chaperone Activity

Hsp70 is most well-known for its role in cytoprotection (Park et al., 2001; Ishii et al., 2003; Sabirzhanov et al., 2012; Kwong et al., 2015), but has also been suggested to regulate cell growth by regulating specific signaling pathways. Hsp70 is an ATP-dependent chaperone with two distinct binding domains: a functional substrate-binding domain and a regulatory nucleotide-binding domain (Blatch and Edkins, 1997). In an ATP-bound state, Hsp70 exists in a relatively open conformation, and has a low affinity for client proteins at the substrate binding domain. Upon ATP hydrolysis, however, Hsp70 undergoes a conformational change that increases its affinity for a given substrate. This cycle is called the chaperone cycle, and is regulated by co-chaperones that promote ATP hydrolysis or nucleotide exchange factors that catalyze the addition of phosphate. One such factor is the B-cell lymphoma 2-associated anthogene-1 (BAG-1), which has been reported to alter the conformation of Hsp70 and reduces its affinity for client proteins at the substrate-binding domain (Bimston et al., 1998). In addition, BAG-1 is known to activate the Raf-1/MAPK/ERK signal transduction cascade, thereby promoting differentiation and proliferation, including in the CNS. Interestingly, Hsp70 has been shown to compete with Raf-1 for binding to BAG-1 *in vitro* (Song et al., 2001), suggesting that Hsp70 could indirectly inhibit cell growth *in vivo*, particularly during stress (Figure 1C). Subsequent studies have confirmed that overexpression of the cytoplasmic isoform of BAG-1, BAG-1s, reduces neurite outgrowth in an Hsp70-dependent manner (Frebel et al., 2007). In the context of neurodevelopment, this negative regulation is consistent with the proposed hypothesis that it may be necessary to temporarily halt energetically expensive processes to ensure cell survival in suboptimal conditions. Although Hsp70’s interactions with BAG-1 seem to inhibit cell growth, interactions with BAG-3 have been reported to promote growth. Consistent with a role in cell survival and growth, several studies have shown that the Hsp70-BAG-3 interaction promotes tumorigenesis through the modulation of specific signal transduction cascades, including those that involve Src and FoxM1 (Colvin et al., 2014; Li et al., 2015). These studies illustrate a co-chaperone-driven role of Hsp70 in cell growth. Like tumorigenesis, neurodevelopment is characterized by high metabolic demands and rapid proliferation. Hsp70, as one of the stress-inducible chaperones, could play a key role in modulating cell growth signaling in such highly demanding conditions.

Hsp90, like Hsp70, is an ATP-dependent chaperone that interacts with numerous client proteins, including transcription factors, tyrosine receptor kinases, and matrix metalloproteinases (Bernstein et al., 2001; Eustace et al., 2004; Wang et al., 2017). Because many of these client proteins are critical to cell growth, proliferation, and migration, numerous researchers are investigating the therapeutic potential of Hsp90 inhibitors as anti-cancer agents. There is growing evidence that the inducible isoform of Hsp90, Hsp90α, also promotes neurite outgrowth. Hsp90 has been long known to be responsible for neurite extension observed in chick embryonic telencephalic and spinal neurons (Ishimoto et al., 1998). However, this observation has received relatively little attention until recent years. Increased Hsp90α synthesis was only recently associated with NGF-mediated neurite outgrowth in the setting of aripiprazole treatment *in vitro* (Ishima et al., 2012). The same authors also showed that silencing of Hsp90α results in attenuated neurite outgrowth, supporting a potential role of Hsp90α in normal neuronal cell growth, particularly through its chaperone activity. This hypothesis is further supported by the observation that inhibition of Hsp90 results in reduced membrane translocation of the growth factor tyrosine receptor kinase A (TrkA) in human acute myeloid leukemia cells (Rao et al., 2010). Interestingly, in this study, inhibition of Hsp90 was associated with an increase in Hsp70, which could reflect a compensatory response similar to what was described by Benitez et al. (2014). Although additional studies are necessary to further understand the specific functions of Hsps in cell growth, collectively, these studies clearly support an important role for Hsps in tumorigenesis, as well as normal development.

## Heat Shock Proteins Participate in Migration and Promote Axon Guidance

### Heat Shock Proteins Participate in Neuronal and Glial Migration

Neuronal migration requires the cell to establish polarity, extend a leading process, survey the extracellular environment, translocate nuclear content, and, finally, advance toward its final destination (Craig and Banker, 1994; Kater and Rehder, 1995). All of these processes rely on remarkably coordinated cytoskeletal dynamics (Mitchison and Kirschner, 1984; Tanaka et al., 2004; Schaar and McConnell, 2005). As mentioned previously, Hsps are well-known to interact with and stabilize cytoskeletal elements (Lavoie et al., 1993b; Sánchez et al., 1994; Weis et al., 2010; Almeida-Souza et al., 2011; Kayser et al., 2013), and, thus, may play a key role in regulating neuronal and glial migration in the developing nervous system. In support of this concept, αB-crystallin was recently reported to promote the formation of mature focal adhesions and slow migration of unstressed C6 glial cells (Shimizu et al., 2016). These new data suggest that αB-crystallin, in addition to its normal chaperone activity, could also regulate cell migration and the proper formation of a neuroglial network. Although αB-crystallin expression in the embryonic brain is mainly limited to glial progenitors (Kida et al., 2010), recent evidence for secretion of αB-crystallin support a separate role on neuronal migration.

**FIGURE 2:**
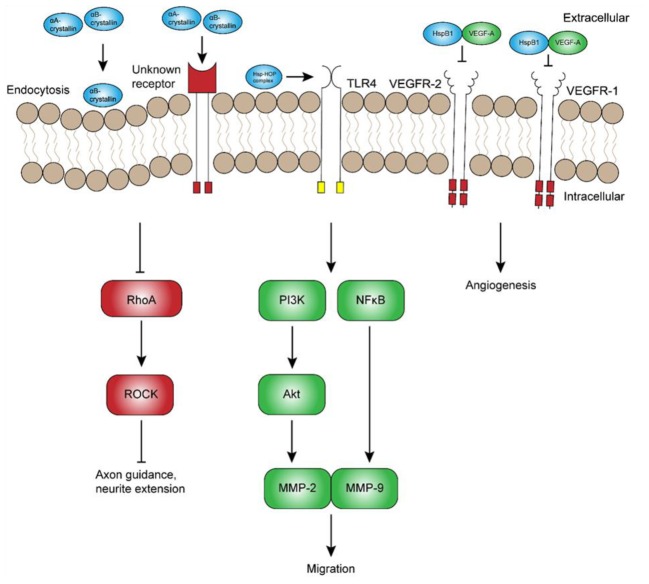
Potential extracellular roles of heat shock proteins in development. Although the exact mechanisms remain unclear, extracellular α-crystallins have been shown to promote axon guidance and neurite extension through inhibition of RhoA-ROCK signaling. Other extracellular Hsps, namely Hsp70 and Hsp90, have been suggested to interact with the Hsp70-Hsp90-organizing protein (HOP) and promote neuronal and glial migration through receptors such as toll-like receptor 4 (TLR4). In contrast, extracellular HspB1 has been shown to inhibit vascular endothelial growth factor (VEGF)-A signaling and angiogenesis by direct inhibition of VEGF-A. Abbreviations: RhoA, Ras homolog gene family, member A; ROCK, Rho-associated protein kinase; PI3K, phosphoinositide 3-kinase; MMP, matrix metalloproteinase; NFκB, nuclear factor κ light chain enhancer of activated B cells.

Stress-inducible Hsps, including Hsp70 and α-crystallins, were recently shown to be secreted into the extracellular milieu, suggestive of novel paracrine and autocrine functions in addition to their classical, intracellular chaperone activity. The first observation of the sort was the fact that Hsp70 can be transferred from heat-stressed squid glia to adjacent neuronal axons (Tytell et al., 1986), suggesting that glia-derived Hsp70 could confer thermotolerance in nearby neurons – a hypothesis that has since been validated *in vitro* (Guzhova et al., 2001). Several Hsps, including HspB1, αA- and αB-crystallin, and Hsp90α, have since been shown to be secreted in exosomes or plasma membrane-derived vesicles (Graner et al., 2007; Wang et al., 2009; Sreekumar et al., 2010; Nafar et al., 2015; Barreca et al., 2017; Ruebsam et al., 2018). On a mechanistic level, HspB1 and Hsp90α, were also shown to be secreted in a phosphorylation-dependent manner, suggesting that secretion of other Hsps may be similarly regulated through phosphorylation of specific residues (Wang et al., 2009; Lee et al., 2012). In the extracellular environment, Hsps have the potential to be taken up by endocytosis or bind to extracellular receptors (Figure 2). Indeed, recent research has confirmed that Hsps can act as a ligand and mediate cell migration in several cell lines (Asea et al., 2002; Barreca et al., 2017).

Hsps are secreted by numerous cell-types, from neurons to glia and endothelial cells (Guzhova et al., 2001; Zhan et al., 2009; Sreekumar et al., 2010; Lee et al., 2012; Ruebsam et al., 2018), and have been implicated as potential contributors to cell migration. Using specific antibodies and inhibitors, Miyakoshi et al. (2017) showed that Hsp70, Hsp90, and co-chaperone Hsp70-Hsp90-organizing protein (HOP) all regulate neuroblast migration from the subventricular zone. Interestingly, Hsp70 and HOP were also found in membrane-associated complexes, suggesting a potential, extracellular role of these chaperones in migration. Although this mechanism has not yet been investigated, one possible explanation is that extracellular Hsps regulate migration through binding to a receptor such as TLR4 (Figure 2), which has been reported to regulate migration of cortical neurons (Moraga et al., 2014). This hypothesis is supported by the finding that extracellular Hsp70 and Hsp90α promote TLR4-dependent migration in mesoangioblasts and glioblastoma cells, respectively (Thuringer et al., 2011; Barreca et al., 2017). While TLR4 is a good candidate, extracellular Hsps could mediate cell migration through several other receptors that remain to be characterized. In any case, significant research is still required to elucidate the role and regulation of extracellular Hsps *in vivo*. In addition to the indirect regulation of cell migration signaling, Hsps may also promote migration by directly serving as guidance cues that promote axon growth and guidance.

### Heat Shock Proteins May Contribute to Axon Guidance

During neurodevelopment, migratory neurons rely on the glial scaffold and extracellular guidance cues to reach their final destination (Marín et al., 2010; Hatten, 1999). As might be expected, Hsps exert numerous chaperone functions within the cell that are critical to transduction of extracellular cues, some of which are necessary for axon guidance (DeGeer et al., 2015). In addition, Hsps have been shown to perform non-classical, extracellular roles that may also promote axon guidance. One of the first studies suggesting this role showed that lens injury-derived factors promote axonal regeneration and pathfinding in a subset of RGCs (Leon et al., 2000). The axons of these RGCs were highly immunopositive for growth associated protein 43, which is known to play a critical role in axon pathfinding during normal neurodevelopment (Strittmatter et al., 1995). Subsequent studies by Fischer et al. (2001, 2008) showed that lens injury-induced axonal regeneration and pathfinding is due mainly to the β-crystallins, a group of non-Hsp crystallins that are similarly found in the vertebrate lens. However, the α-crystallins also appeared to exert some effect, although this effect was not as strong as that of β-crystallins. As discussed in a previous section of this review, other studies have since shown that the co-purified aggregates containing αA- and αB-crystallins can promote neurite extension *in vitro*. Furthermore, both αA- and αB-crystallin are secreted in vesicles (Sreekumar et al., 2010; Ruebsam et al., 2018), raising the possibility that these chaperones could mediate numerous, novel functions, such as axon guidance, through endocytic or receptor-mediated pathways that remain to be discovered. Given that αB-crystallin is not highly expressed in developing telencephalic neurons (Kida et al., 2010), it most likely promotes axon outgrowth and guidance through a paracrine mechanism, similar to the β-crystallins (Fischer et al., 2001, 2008).

## Heat Shock Proteins Contribution to the Formation of the Retinal Neurovascular Unit

### Heat Shock Proteins Regulate Angiogenesis

Successful neurodevelopment depends on the anatomic and physiologic integration of neurons, glia, and the underlying vascular network. This functional unit has been termed the neurovascular unit, and its importance is reflected in congenital conditions such as persistent hyperplastic primary vitreous (Kurihara, 2016), as well as neurodegenerative diseases such as Alzheimer’s disease and diabetic retinopathy (Iadecola, 2004; Gardner and Davila, 2017). HspB1 is strongly associated with the first endothelial cells in the developing CNS (Loones et al., 2000), suggestive of a potential role of Hsps in endothelial cell proliferation. In the past decade, several studies have implicated Hsps as novel regulators of specific angiogenic factors, and, therefore, these chaperones may also regulate formation of the neurovascular unit. One such factor is vascular endothelial growth factor A (VEGF-A), an endothelial cell-specific mitogen and potent agonist of angiogenesis (Kim et al., 1993; Ferrara et al., 2003). Kase et al. (2010) clearly demonstrated the necessity of αB-crystallin in two murine models of angiogenesis, namely oxygen-induced retinopathy (OIR) and laser-induced choroidal neovascularization (CNV). In the OIR model, αB-crystallin^−/−^ mice exhibited significantly reduced VEGF-A protein levels and angiogenesis compared to wild-type mice, particularly in the highly vascular inner plexiform layer of the retina. Using a laser-induced CNV model, the same authors showed that pharmacologic inhibition of the proteasome restores VEGF-A protein levels in αB-crystallin^−/−^ mice, suggesting that αB-crystallin protects VEGF-A from proteasomal degradation. Because αB-crystallin is induced in response to stress, this chaperone could contribute to hypoxia-mediated angiogenesis during neurodevelopment, particularly through the stabilization of VEGF.

The pro-angiogenic effect of αB-crystallin appears to be balanced at least in part by HspB1 and αA-crystallin, both of which have been reported to attenuate angiogenesis. HspB1 expression during CNS development is not only associated with endothelial cell migration and differentiation, but also secreted from endothelial cells into the extracellular environment, where it participates in a negative feedback loop and directly inhibits VEGF-mediated angiogenesis and tumorigenesis (Figure 2; Lee et al., 2012). Subsequent experiments indicated that HspB1 secretion from endothelial cells is inhibited by a feedback mechanism involving VEGF-mediated phosphorylation. Similarly, others have shown that the exogenous application of αA-crystallin inhibits corneal neovascularization (Zhu et al., 2012). In the context of neurodevelopment, one group has shown that αA-, as well as αB-crystallin, undergoes a marked increase in mRNA expression between P17 and P21 in the OIR model (Shi et al., 2015). Unlike αB-crystallin, however, increased expression of αA-crystallin is not associated with an obvious change in Hsp synthesis detectable by immunofluorescence, suggesting that chaperone-mediated regulation of angiogenesis may occur mainly through αB-crystallin.

Recent research has also uncovered a critical role of βA3/A1-crystallin, a member of the β-crystallin family, in the formation of the neurovascular unit. Although the β-crystallins are not Hsps, these proteins were similarly first identified in the vertebrate lens, and are now understood to be equally necessary in non-lenticular tissues, especially in the retina (Zhang et al., 2005). One group has performed extensive research on the role of βA3/A1-crystallin in retinal vasculature development, and has shown that βA3/A1-crystallin mutations are associated with abnormal structure and function of retinal astrocytes, leading to persistent hyperplastic primary vitreous (Sinha et al., 2008, 2012; Valapala et al., 2015; Zigler et al., 2016). While the α- and β-crystallins are generally considered to be evolutionarily distinct, it is possible these proteins share some functional similarities that remain to be discovered.

In contrast to the small Hsps, relatively little research has been performed on the large Hsps in the context of angiogenesis. Although Hsp90 has not been demonstrated to interact directly with VEGF, Hsp90 is known to interact with hypoxia-inducible factor-1α as well as VEGF receptors 1 and 2 (Minet et al., 1999; Park et al., 2008), potentially modulating VEGF activity and angiogenesis. Using the OIR model, a study showed that Hsp90 inhibitors reduce hypoxia-inducible factor 1α-mediated angiogenesis in the hypoxic mouse retina (Jo et al., 2014). These conditions enhance the hypoxia-driven formation of new blood vessels during normal embryogenesis, suggesting that Hsps could contribute to the formation of the neurovascular network during normal development. While the relative contribution of each Hsp remains to be determined, Hsps likely play a key role in the complex balance of pro- and anti-angiogenic processes that result in the normal vascular network.

## Recent Controversies and Developments

### Heat Shock Proteins Are an Important Determinant of Cell Survival

This review has focused on the neuroprotective role of Hsps in the setting of developmental or physiologic stress and in the absence of stress, but some have suggested that overexpression of Hsps could be detrimental to cell survival (Narayanan et al., 2006; Richter-Landsberg et al., 2010). At least one study has suggested that αB-crystallin is associated with amyloid-β accumulation and may contribute to neurodegenerative diseases (Narayanan et al., 2006). However, many more studies have shown that αA- and αB-crystallin, as well as Hsp27, inhibit the formation of amyloid-β and neurofibrillary tangles (Waudby et al., 2010; Shammas et al., 2011; Hochberg et al., 2014; Cox et al., 2018). This apparent controversy is nicely summarized by Kannan et al. (2012), who recently reviewed the role of α-crystallins in neurodegenerative disease and discuss the potential therapeutic benefit of αB-crystallin, in particular. Additional evidence for the neuroprotective role of Hsps can be seen in different models of retinal neurodegeneration, in which αA- and αB-crystallin knockouts have shown markedly enhanced pathologic findings compared to wild-type controls (for review, see Fort and Lampi, 2011). Pharmacologic studies, too, support a neuroprotective role of Hsps. One of the most well-known examples is the pharmacologic agent arimoclomol, which upregulates the heat shock response and protects against neurodegenerative diseases such as amyotrophic lateral sclerosis and retinitis pigmentosa (Kieran et al., 2004; Parfitt et al., 2014). Although this drug recently completed phase II clinical trials (Benatar et al., 2018), further research will be required to determine whether a therapeutic benefit exists. In all likelihood, Hsps are one of the key factors that can tip the balance between survival and apoptosis.

## Concluding Remarks

There is an accumulation of evidence to suggest that Hsps play a critical role in neurodevelopment; however, the exact role of these chaperones remains largely unexplored. Hsps are expressed early in neurodevelopment and exhibit a specific spatio-temporal pattern that coincides with neuronal differentiation. Furthermore, recent research has highlighted the potential regulatory role of these chaperones in key events, including neurite extension, migration, and axon guidance. Whether these chaperones perform similar functions *in vivo* remains to be seen and will require additional studies, but represents an exciting possibility not only in neurodevelopment, but also in anti-cancer therapy and the treatment of neurodegenerative diseases.

## Author Contributions

DM wrote the manuscript, produced the supporting table and figures, and obtained funding. PF obtained funding, directed the writing, and edited the manuscript.

## Conflict of Interest Statement

The authors declare that the research was conducted in the absence of any commercial or financial relationships that could be construed as a potential conflict of interest.
